# Genetic diversity of Trichomonads from Milu deer (*Elaphurus davidianus*) in China

**DOI:** 10.1051/parasite/2025015

**Published:** 2025-04-09

**Authors:** Yilei Zhang, Zhouchun Li, Xinglong Song, Guodong Xiao, Lingru He, Jiade Bai, Zhenyu Zhong, Lijie Tian, Yan Chang, Zhuang Li, Qingyun Guo, Congshan Yang, Qingxun Zhang

**Affiliations:** 1 College of Veterinary Medicine, Anhui Agricultural University Hefei Anhui Province 230036 China; 2 Beijing Milu Ecological Research Center Beijing 100076 China; 3 College of Life Science, Anhui Agricultural University Hefei Anhui Province 230036 China; 4 Biotechnology Center of Anhui Agricultural University Hefei Anhui Province 230036 China

**Keywords:** Milu deer, Trichomonads, Parabasalia, China

## Abstract

The Milu deer, or Père David’s deer (*Elaphurus davidianus*), a rare endemic species in China, represents a case of successful reintroduction of a species previously considered extinct in the wild. Trichomonads, protozoan symbionts capable of infecting vertebrates, are transmitted via the fecal-oral route; they are a subgroup of Parabasalia and include some pathogenic species that pose zoonotic risks. Until now, data on the diversity and prevalence of trichomonads in Chinese Milu deer have not been reported. To better understand the colonization status of trichomonads, fecal samples from 112 Milu deer across five nature reserves in China were collected. The ITS-1/5.8S/ITS-2 sequences were amplified using PCR to investigate the colonization rate of trichomonads and to assess evolutionary relationships and genetic characteristics through phylogenetic analysis. An occurrence of 38.39% was recorded in Milu deer, with sample collection sites (OR = 55.159, 95% CI = 3.166–961.113, *p* = 0.006), high relative humidity and average annual rainfall (OR = 11.675, 95% CI = 1.747–77.781, *p* = 0.011) identified as significant risk factors for trichomonads colonization. Undescribed trichomonads from four genera were identified, including *Simplicimonas* spp., *Hypotrichomonas* spp., *Hexamastix* spp., *and Tetratrichomonas* spp. To our knowledge, this is the first study to report on trichomonads in Milu deer in China. This study aims to enhance understanding of trichomonad colonization and associated risk factors, providing scientific guidance for the *ex-situ* conservation of Milu deer.

## Abstract (fr)

Le cerf milu, ou cerf du Père David (*Elaphurus davidianus*), espèce rare et endémique en Chine, représente un cas de réintroduction réussie d’une espèce auparavant considérée comme éteinte à l’état sauvage. Les Trichomonadida, symbiotes protozoaires capables d’infecter les vertébrés, se transmettent par voie fécale-orale; ils constituent un sous-groupe des Parabasalia et comprennent certaines espèces pathogènes présentant un risque zoonotique. Jusqu’à présent, aucune donnée sur la diversité et la prévalence des Trichomonadida chez le cerf milu n’a été rapportée. Afin de mieux comprendre l’état de colonisation des Trichomonadida, des échantillons fécaux ont été prélevés sur 112 cerfs répartis dans cinq réserves naturelles de Chine. Les séquences ITS-1/5.8S/ITS-2 ont été amplifiées par PCR pour étudier le taux de colonisation des Trichomonadida et évaluer les relations évolutives et les caractéristiques génétiques par analyse phylogénétique. Une occurrence de 38,39 % a été enregistrée chez le cerf milu, les sites de collecte d'échantillons (OR = 55,159, IC à 95 % = 3,166 – 961,113, *P* = 0,006) et une humidité relative élevée et des précipitations annuelles moyennes élevées (OR = 11,675, IC à 95 % = 1,747 – 77,781, *P* = 0,011) étant identifiés comme des facteurs de risque significatifs pour la colonisation par les Trichomonadida. Des Trichomonadida non décrits appartenant à quatre genres ont été identifiés, dont *Simplicimonas* spp., *Hypotrichomonas* spp., *Hexamastix* spp. et *Tetratrichomonas* spp. À notre connaissance, il s’agit de la première étude à signaler la présence de Trichomonadida chez le cerf milu en Chine. Cette étude vise à améliorer la compréhension de la colonisation par les Trichomonadida et des facteurs de risque associés, fournissant ainsi des orientations scientifiques pour la conservation ex situ du cerf milu.

## Introduction

The Milu deer, or Père David’s deer, *Elaphurus davidianus* Milne-Edwards, 1866, the sole species within its genus, is classified under the order Artiodactyla, family Cervidae, and subfamily Cervinae [21, 47]. After a three-step strategy of population recovery, *ex-situ* conservation, and rewilding release, the population has grown to over 12,000 individuals across more than 90 populations [30]. The re-introduction and revitalization of the Milu deer is a global conservation triumph, demonstrating substantial success in conservation and reintroduction efforts, and exemplifying best practices in biodiversity conservation for United Nations Ecosystem Restoration projects [51]. Nevertheless, increasing population densities have exacerbated conflicts between the deer and their habitats, particularly as parasitic co-colonizations have contributed to a higher incidence of diseases, thereby limiting the healthy development of the individual [13, 23, 45, 48–50].

Trichomonads, a diverse group of anaerobic flagellated protozoa belonging to Parabasalia, are characterized by the presence of hydrogenosomes instead of mitochondria and the unique morphology of their flagella: flagellar apparatus, paraflagellates, and paraflagellar filaments [1, 7, 8]. Of the approximately 500 described Parabasalia species, the vast majority are endosymbionts, commensals, or parasites of various vertebrates (especially termites) and invertebrates, typically residing in the distal portion of the digestive tract [8]. Recently, six free-living species have been identified, mainly from freshwater and marine sediments [11]. Historically, the phylum parabasalia was divided into two groups based on morphological characteristics; however, the current taxonomic system recognizes six classes: Trichomonadea, Hypotrichomonadea, Tritrichomonadea, Cristamonadea, Spirotrichonymphea, and Trichonymphea [8, 9]. Although some species are harmless, several are pathogenic and of significant medical and veterinary importance [38], including *Trichomonas vaginalis*, which infects the genitourinary tract [28]; *Trichomonas tenax*, which is parasitic in the oral cavity [16]; and *Pentatrichomonas hominis* and *Dientamoeba fragilis*, which are present in the gastrointestinal tract and represent major human pathogens [26, 39]. Additionally, several species such as *Trichomonas gallinae*, *Tetratrichomonas gallinarum*, and *Histomonas meleagridis*, are avian pathogens [2, 5, 52], and *Tritrichomonas foetus* is known to cause abortion in pregnant cows [37].

Recent studies have demonstrated that trichomonads can parasitize non-unitary sites in hosts, adapt to new hosts, and pose a zoonotic risk [4, 6, 19, 27]. Consequently, there is growing interest in studying trichomonads to identify species that may exhibit zoonotic characteristics [10]. To date, little information exists on the occurrence and genetic diversity of trichomonads in Milu deer. Therefore, the present study aimed to determine the occurrence, risk factors, and genetic diversity of trichomonads in Milu deer, providing scientific guidance for *ex-situ* conservation of Milu deer.

## Materials and methods

### Ethical approval

This study adhered to the Guide for the Care and Use of Laboratory Animals set forth by the Ministry of Health, China. The protocol received approval from the Research Ethics Committee of the Anhui Agricultural University (number AHAUB2022019). Throughout the sample collection process, care was taken to ensure that the animals were not disturbed and stressed.

### Study sites, sample collection, and sample processing

The present study was conducted at the following five sampling sites: (1) Beijing Nanhaizi Milu Park National Natural Reserve, located in the northern part of the North China Plain and the southern edge of the Yanshan Mountains; (2) Hebei Upstream of Luanhe River National Nature Reserve, located at the confluence of the Yinshan Mountains, the Daxingan Mountains and the remnants of the Yanshan Mountains; (3) Hubei Shishou Milu National Nature Reserve, located at the geographical center of the middle reaches of the Yangtze River; (4) Jiangsu Dafeng Milu National Nature Reserve, located on the coast of the Yellow Sea, east of Jiangsu Province, south of Yancheng City, and east of the Yellow Sea; and (5) Inner Mongolia Daqingshan National Nature Reserve, located in the Yinshan Mountains of the Inner Mongolia Autonomous Region. The data used to evaluate the possible risk factors associated with colonization were recorded, including the geographic coordinates, annual mean temperature, relative humidity, average annual rainfall, altitude, and number of individuals per hectare in these nature reserves (Table 1).

**Table 1:** Summary of characteristics of the five Milu nature reserves in China.

	Nanhaizi Milu Park, Beijing	Upstream of Luanhe River Milu Nature Reserve, Hebei	Shishou Milu Nature Reserve, Hubei	Dafeng Milu Nature Reserve, Jiangsu	Daqingshan Milu Nature Reserve, Inner Mongolia
Geographic coordinates	39°46’N, 116°26’E	41°47’N, 116°51’E	29°49’N, 112°33’E	33°5’N, 120°49’E	41°53’N, 120°24’E
Annual mean temperature (°C)	13.1	2.8	16.5	14.1	4.2
Relative humidity (%)	65	60	80	80	60
Average annual rainfall (mm)	600	500	1200	1000	400
Altitude (m)	31.5	750.0–1829.0	32.9–38.4	1.0–2.0	1030.0–2338.0
Floor space (hm^2^)	55	50637	1567	78000	26989
Feeding status	Semi-free	Semi-free	Free	Free	Free
Number of animals	190	35	2500	3917	36
Vegetation condition	Aquatic vegetation, thicket, arbor forests, etc.	Sandy forests, alpine meadows, coniferous forests, deciduous broad-leaf forests lake vegetation, etc.	Swamped meadow, lake vegetation, swamp vegetation, shoal grassland and dry willow thickets, etc.	Salt meadow, swamp vegetation, aquatic vegetation, deciduous broad-leaf forest and sparse shrubwood, etc.	Grassland meadows, deciduous broad-leaf forest, shrub-wood, brush and grass, etc.

A total of 112 fecal samples were collected from the Milu deer National Nature Reserve from September 2022 to December 2023. These specimens were from five localities (Table 2), including Beijing Nanhaizi (*n* = 46), Hebei Upstream of Luanhe River (*n* = 13), Hubei Shishou (*n* = 12), Jiangsu Dafeng (*n* = 15), and Inner Mongolia Daqingshan (*n* = 26). Fresh and relatively intact feces were collected immediately after Milu deer defecation. To ensure standardization of the fecal sample collection process and to reduce potential contamination between samples, all fecal samples were collected with the assistance of experienced Nature Reserve staff. During sample collection, only the middle layer of feces was collected and stored separately in an insulated sampling box. All fecal samples were submitted to the laboratory under ambient storage and examined by light microscopy within 4 h. Following this, the remaining portions of all the samples were then immediately stored at −80 °C until DNA was extracted.

**Table 2:** Occurrence of trichomonad colonization in Milu deer. A potential risk factor for colonization was considered when *p* < 0.05 and OR > 1.

Province	No. of samples	Number of colonizations (*n*)/Colonization rate (%)	OR	95% CI	*p*
Beijing	46	12/26.08	0.398	0.176–0.902	0.027*
Hebei	13	6/46.15	1.436	0.448–4.600	0.542
Hubei	12	12/100	55.159	3.166–961.113	0.006**
Jiangsu	15	8/53.33	2.024	0.677–6.056	0.207
Inner Mongolia	26	5/19.23	0.301	0.104–0.872	0.027*
Total	112	43/38.39			

The *p*-values were calculated by comparing the sum of individual groups with other groups. *p < 0.05, **p < 0.01.

### DNA extraction and PCR amplification

Genomic DNA was extracted from approximately 200 mg of each fecal sample using a TIANamp Stool DNA Kit (Beijing TransGen Biotech Co., Ltd., Beijing, China), according to the manufacturer’s instructions. Genomic DNA was eluted into 50 μL of elution buffer and stored at −40 °C until PCR amplification.

PCR was amplified, targeting an approximately 350 base pair (bp) fragment in the ITS-1/5.8S/ITS-2 sequence to determine the trichomonad species. The primers were TFR1 (5′-TGCTTCAGTTCAGCGGGTCTTCC-3′) and TFR2 (5′-CGGTAGGTGAACCTGCCGTTGG-3′) [11, 35].

The amplification process was performed in 25 μL volumes, including 1 μL of template DNA or primary PCR product, 2.5 μL 10 × KOD-Plus PCR buffer, 2.5 μL dNTPs (2 nM), 1.5 μL MgSO_4_ (25 nM), 0.5 μL of each primer (25 nM), 16 μL double-distilled water, and 0.5 μL KOD-Plus amplification enzyme (1 unit/μL) (ToYoBo Co., Ltd., Osaka, Japan). There was an initial denaturation at 94 °C for 5 min, 35 cycles at 94 °C for 45 s, 55 °C for 45 s, 72 °C for 30 s, and a final extension at 72 °C for 5 min in PCR amplification. The positive (DNA from the PCR-positive snake fecal samples) and negative (1 μL double-distilled water replaced 1 μL template DNA) controls were used in each PCR amplification. The reactions were performed in a thermocycler (Applied Biosystems, Foster City, CA, USA). The final products from the amplification were analyzed by electrophoresis on 1% agarose gel, by staining with ethidium bromide and observed under UV light.

### Cloning

Positive PCR amplification products sequenced in bidirectional displayed double peaks. To confirm that the sequencing reaction from the PCR product was not affected by the presence of contaminating PCR products, PCR products were purified using a FastPure Gel DNA Extraction Mini Kit (Vazyme Biotech Co., Ltd., Nanjing, China), according to the manufacturer’s instructions. Positive products were cloned into a 5min^TM^ TA/Blunt-Zero Cloning Kit (Vazyme Biotech Co., Ltd.) and used to transform *Escherichia coli* DH5α cells. Clones were selected on LuriaBertani (LB) agar supplemented with 50 μg/mL of ampicillin.

### Sequence and phylogenetic analysis

All positive clones were sequenced from the vector and identified the species of trichomonads. These recombinant clones were sequenced unidirectionally at Tsingke Biotech Co., Ltd., Beijing, China. The primers used for sequencing were M13F (5′-GTAAAACGACGGCCAGT-3′) and M13R (5′-CAGGAAACAGCTATGAC-3′). Nucleotide sequences were assembled with ChromasPro2.1.6 (https://www.technelysium.com.au/ChromasPro.html), edited using BioEdit 7.1.3 (https://www.mbio.ncsu.edu/BioEdit/bioedit.html). Nucleotide sequences were aligned with the reference sequences from GenBank using ClustalX 2.1 software (https://www.clustal.org). Phylogenetic relationships among the species were evaluated using neighbor-joining analyses in MEGA 11 (https://www.megasoftware.net/), with branch reliability assessed by 1,000 bootstrap replicates. A phylogenetic tree representing the flagellated protozoa was then constructed. Nucleotide sequences generated in the study were submitted to GenBank under accession numbers PP829134–PP829172 and PP829309–PP829318. Three sequences failed to upload to NCBI and are presented as Supplementary File 1.

### Statistical analysis

The chi-square (χ^2^) test, conducted using IBM SPSS Statistics 22 software (International Business Machines Corp., New York, NY, USA), was used to compare differences in trichomonad colonization rates across different living conditions of Milu deer. Odds ratios (ORs) with 95% confidence intervals (CIs) were used to identify the risk factors associated with the occurrence of these pathogens in Milu deer. Differences were considered statistically significant at *p* < 0.05.

## Results

### Occurrence of and risk factors for trichomonads in random sampling

Among the 112 fecal samples collected, PCR testing made it possible to identify 43 as positive for trichomonads (Table 2). The occurrence ranged from 19.23% to 100% in different provinces. Data analysis revealed that Milu deer in Hubei (100%, 12/12) had a significantly higher detection rate compared to those in Beijing (26.08%, 12/46; χ^2^ = 21.435, *p* < 0.001), Inner Mongolia (19.23%, 5/26; χ^2^ = 21.665, *p* < 0.001), Hebei (46.15%, 6/13; χ^2^ = 8.974, *p* = 0.003), and Jiangsu (53.33%, 8/15; χ^2^ = 7.560, *p* = 0.006). Significant differences in colonization rates observed between Jiangsu (53.33%, 8/15) and Inner Mongolia (19.23%, 5/26; χ^2^ = 5.109, *p* = 0.024); however, differences with Beijing (26.08%, 12/46; χ^2^ = 3.811, *p* = 0.051), and Hebei (46.15%, 6/13; χ^2^ = 0.144, *p* = 0.705) were not statistically significant. In addition, there were no significant impacts on the colonization rates in Beijing (26.08%, 12/46), Hebei (46.15%, 6/13; χ^2^ = 1.925, *p* = 0.165), and Inner Mongolia (19.23%, 5/26; χ^2^ = 0.433, *p* = 0.511).

**Figure 1:**
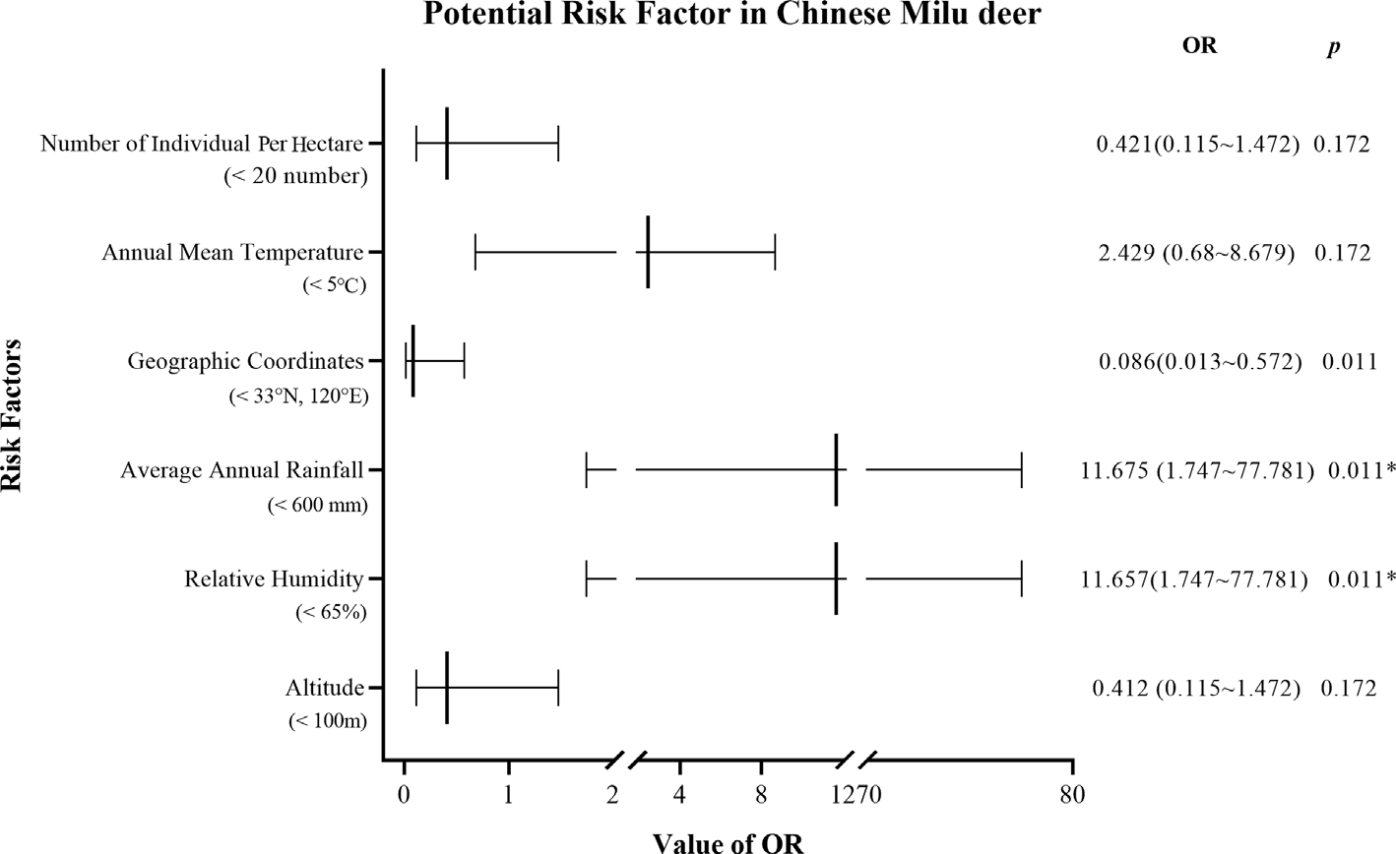
Association between trichomonad colonization and distribution of five Milu nature reserves in China, indicated by univariate analyses of data from this study. A potential risk factor for colonization was considered when *p* < 0.05 and OR > 1. *p < 0.05.

Furthermore, OR analysis revealed that Milu deer in Hubei exhibited a significantly higher risk of trichomonad colonization, with an occurrence of 100% (OR = 55.159, 95% CI = 3.166–961.113, *p* = 0.006), indicating notable vulnerability due to varying localities (Table 2). Results of the logistic regression analysis indicated that trichomonad colonization was not significantly associated with geographic coordinates, annual mean temperature, altitude, and number of individuals per hectare in the four Milu nature reserves (*p* > 0.05). However, there was an apparent association between trichomonad colonization with high relative humidity and average annual rainfall (OR = 11.675, 95% CI = 1.747–77.781, *p* = 0.011) (Figure 1).

### Distribution of trichomonad species

**Figure 2:**
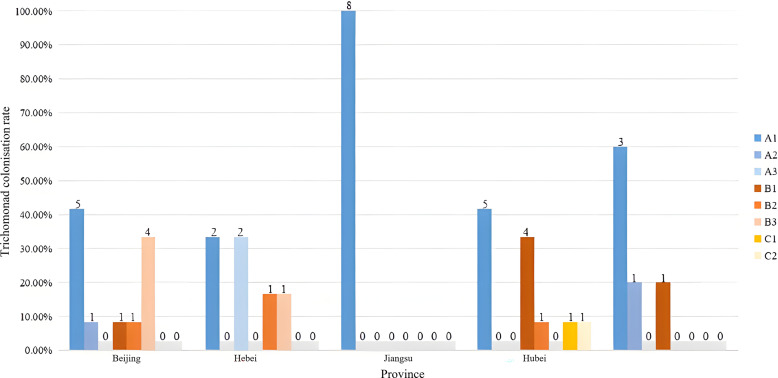
Occurrence of trichomonad pathogens in Milu deer. Vertical columns show co-colonization rates in the same province. Single pathogen colonization, A1: *Simplicimonas*, A2: *Hypotrichomonas*, A3: *Hexamastix*. Co-colonizations with two pathogens, B1: *Simplicimonas* & *Hypotrichomonas*, B2: *Simplicimonas* & *Hexamastix*, B3: *Simplicimonas* & *Tetratrichomonas*. Co-colonizations with three pathogens, C1: *Simplicimonas*, *Hypotrichomonas*, & *Hexamastix*, C2: *Simplicimonas*, *Hypotrichomonas*, & *Tetratrichomonas*.

Among the 43 positive cloning products, four genera and 52 sequences from trichomonads were successfully identified via sequence analysis. Colonizations ranged from single to triple pathogen co-colonizations (Figure 2). Notably, 27 Milu deer were positive for single pathogen colonization, with occurrence rates for *Simplicimonas* spp. at 53.49%, and both *Hypotrichomonas* spp. and *Hexamastix* spp. at 4.65% each. Co-colonizations involving two pathogens occurred in 32.56% of cases (14/43), with combinations of *Simplicimonas* spp. and *Hypotrichomonas* spp. at 13.95%, *Hexamastix* spp. at 6.98%, and *Tetratrichomonas* spp. at 11.63%. Additionally, co-colonizations with three pathogens – *Simplicimonas* spp., *Hypotrichomonas* spp., and *Hexamastix* spp. – were observed in 2.33% of cases (1/43), similar to the co-colonization rate of *Simplicimonas* spp., *Hypotrichomonas* spp., and *Tetratrichomonas* spp. at 2.33% (1/43).

Across the sampling locations, only *Simplicimonas* spp. colonizations were found in the Milu deer in Jiangsu. However, five different combinations of four-pathogen colonizations occurred in Beijing and Hubei, respectively. Four types of colonization forms involving combinations of three pathogens, excluding *Hypotrichomonas* spp., were observed in Hebei. In Inner Mongolia, pathogens associated with *Simplicimonas* spp. and *Hypotrichomonas* spp. presented three different modes of colonization (Figure 1).

### Genetic and phylogenetic analysis

**Figure 3:**
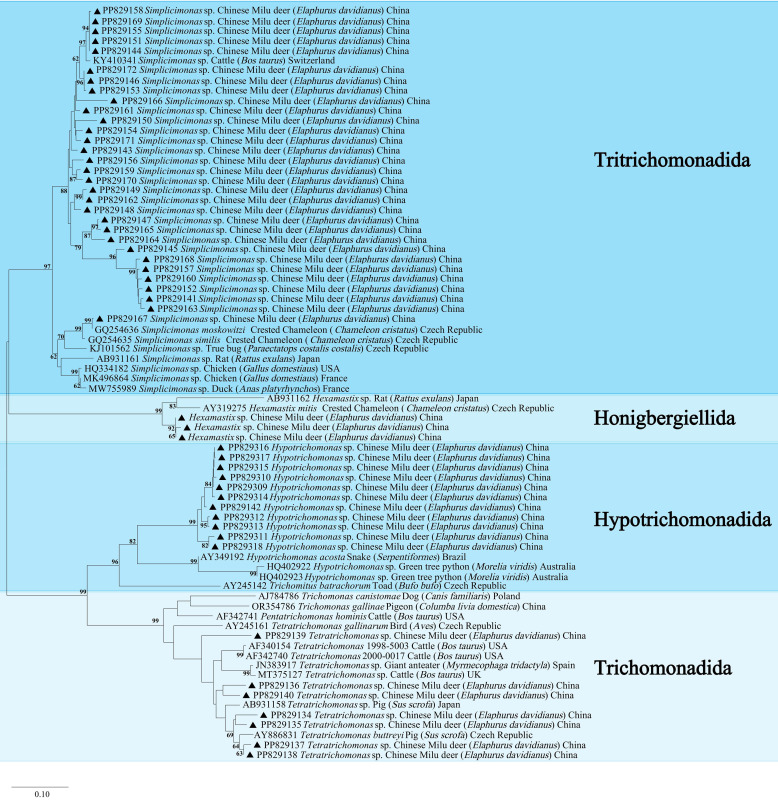
Phylogenetic analysis of trichomonads based on the ITS1-5.8S rRNA-ITS2 gene. Sequences were retrieved from GenBank, aligned using ClustalW, and analyzed using MEGA 11 software. The neighbor-joining method was used to construct the trees from the Kimura-2-parameter model. Branch numbers represent percent bootstrapping values from 1,000 replicates, with values of more than 60% shown in the tree. The species identified in this study are indicated by ▲.

No ITS-1/5.8S/ITS-2 sequences from trichomonads in Milu deer are available in GenBank; thus, phylogenetic analysis was conducted using comparable sequences (Figure 3). The analysis confirmed the identity of *Hypotrichomonas* spp., which shared 82.95–89.37% nucleotide identity with a strain isolated from a snake in Brazil (AY349192). The *Tetratrichomonas* spp. sequences were 89.47–96.01% identical to a strain isolated from a pig (AY886831). Additionally, the sequences of *Simplicimonas* spp., sharing 93.81–96.43% nucleotide identity, clustered in the clade with a strain isolated from cattle (KY410341). Furthermore, PP829141 showed a 99.67% nucleotide identity with a sequence from a chamaeleon (GQ254636). Moreover, three *Hexamastix* spp. sequences, not yet assigned NCBI accession numbers, exhibited 87.07–87.39% nucleotide similarity with a sequence from a rat (AB931162) and 91.97–92.36% with a sequence from a chamaeleon (AY319275). In order to rationalize Figure 3, we present a more general phylogenetic analysis tree covering a wider range of trichomonads as Supplementary File 2.

## Discussion

Traditionally, microscopic examination of trichomonas in feces has been considered the gold standard [31–33]. However, differentiating between trichomonas species with similar morphological characteristics is often impractical in field conditions when examining Milu deer feces. Therefore, more sensitive PCR methods have been adopted for the detection and identification of trichomonas species. Although many trichomonads function as harmless intestinal symbionts, others, such as *P. hominis*, *T. gallinae*, *T. gallinarum*, and *T. foetus*, can cause significant diseases in livestock and are critically important in veterinary medicine [16, 26, 28, 38, 39]. Identifying the specific trichomonad species in Milu deer is therefore crucial and contributes valuable insights for the *ex-situ* conservation of this species.

In the current work, we investigated the occurrence of trichomonad in Milu deer via amplification of the ITS1-5.8S rRNA-ITS2 gene. The overall colonization rate in Milu deer was 38.39% (43/112). Globally, trichomonas infections are prevalent in various hosts. In pigs, the infection rates were reported at 52.08% (100/192) in Austria [40], 43.04% (68/158) in Changchun, and 47.4% (237/500) in Anhui, China [24, 31]. For non-human primates (NHPs), the rates were 46.7% (28/60) in North China [34], 27.6% (45/163) among gorillas in the Central African Republic [41], 68.6% (48/70) in wild chimpanzees in Uganda [43], and 31.3% (5/16) in southeast Brazil [15]. These findings underscore the extensive and significant occurrence of trichomonads across diverse host populations. Thus, detecting trichomonad colonization in Milu deer is significant, as it further expands the host range of trichomonads.

The occurrence of trichomonad colonization varied significantly across different nature reserves in this study. In Hubei, the occurrence (100%, 12/12) and risk of trichomonad colonization (OR = 55.159, 95% CI = 3.166–961.113, *p* = 0.006) were markedly higher than in other regions (Table 2). This variation in colonization rates may be attributable to the environmental conditions [36, 52]. Factors such as the higher relative humidity and average annual rainfall in Hubei likely contribute to elevated colonization rates. A logistic regression analysis of the distribution summaries of the five nature reserves confirms that the high humidity and average annual rainfall (OR = 11.675, 95% CI = 1.747–77.781, *p* = 0.011) in Hubei probably contributed to the high colonization rate of Milu deer (Figure 1). Additionally, the size and density of Milu deer herds in nature reserves could facilitate the accumulation of parasites in the environment [46]. Stagnant water in the marsh contaminates the environment. Milu deer become carriers of parasites due to the contaminated environment, spreading the colonization through fecal-oral transmission and increasing the risk of trichomonad colonization. Finally, the limited number of samples collected might also have influenced the observed differences in trichomonad occurrence across various regions.

Four species of trichomonads were found in Milu deer (Figure 2). Although some trichomonads may have been traditionally viewed as parasites, recent studies suggest that they may also act as mutualists, contributing to the host’s health and gut homeostasis. For instance, the genus *Tritrichomonas* can help regulate the gut microbiome by promoting microbial diversity or enhancing nutrient absorption, benefiting the host in a symbiotic manner [42]. This mutualistic relationship could lead to improved digestive efficiency, better immune function, and protection against other pathogens by maintaining a stable gut microbiota.

*Simplicimonas* spp., members of the class Tritrichomonadea, are typically considered commensals of the intestinal tract due to their non-pathogenic nature [14, 20]. However, this genus has been found in a variety of hosts including livestock, birds, rodents, and reptiles, sometimes in association with *T. gallinarum*. Although *Simplicimonas* spp. are generally regarded as low pathogenicity species that exhibit host specificity among avians, they occasionally present as granulomatous disease [29]. Given the widespread distribution of *Simplicimonas* spp. in the Milu deer, it is plausible that these organisms may have a similar role as commensals or mutualists in this host species. Further research should aim to clarify the functional roles of *Simplicimonas* spp. in the gut ecosystem of the Milu deer and examine whether their presence contributes to positive outcomes, such as improved gut health or metabolic processes.

*Tetratrichomonas* spp. comprises approximately ten valid species and is a common parasite that readily transmits, infecting various vertebrates, including humans, ruminants, birds, rodents, and NHPs under favorable conditions [3, 18, 37]. This genus has been identified as an opportunistic pathogen. Previous studies have indicated that an overgrowth of *Tetratrichomonas* spp*.* in cattle can lead to diarrhea, causing significant economic losses in domestic animals and adversely affecting the health of wild animals [3, 17]. Consequently, it is essential to monitor Milu deer for *Tetratrichomonas* spp. regularly to prevent and control potential diseases.

*Hypotrichomonas* spp., a relatively obscure genus within the class Hypotrichomonadida, has been reported in NHPs, reptiles, and rodents [12]. Similarly, species of *Hexamastix*, belonging to the class Honigbergiellida, have been known to infect reptiles and rodents [25, 44]. In this study, we detected both *Hypotrichomonas* spp. and *Hexamastix* spp. in Milu deer, thereby broadening their known host range. However, the scant information on the pathogenic roles of *Hypotrichomonas* spp. and *Hexamastix* spp. complicates the determination of their effects in these hosts. Further research is required to understand their life cycles and pathogenic potential.

*Hypotrichomonas* spp. and *Hexamastix* spp., detected in Milu deer, clustered in a separate clade rather than with the reference sequences, while *Simplicimonas* spp. and *Tetratrichomonas* spp. predominantly aligned with the reference sequences (Figure 3). This divergence may stem from the limited host range of *Hypotrichomonas* spp. and *Hexamastix* spp., as current studies have identified only a few strains. Conversely, *Simplicimonas* spp. and *Tetratrichomonas* spp. are known to infect a wide variety of hosts [3, 14, 18, 20]. Notably, none of the sequences were 100% homologous with those in the NCBI during comparisons, suggesting distinct genetic profiles of trichomonads in Milu deer compared to those in other hosts. Interestingly, phylogenetic analysis revealed that *Tetratrichomonas* spp. and *Hypotrichomonas* spp. from the same nature reserves clustered in a single clade. This may indicate variability in Milu deer intestinal trichomonads across nature reserves. Consequently, more comprehensive evolutionary analyses and studies on trichomonads in Milu deer are imperative to enhance the accuracy of parasitism detection and inform the development of effective prevention and treatment strategies.

Furthermore, previous studies demonstrated the existence of genetically distinct mixed infections of *T. gallinae* in individual animals [22]. In our study, based on the species differences of trichomonads, mixed colonizations were considered to be present in Milu deer. Consequently, management of living conditions and regular monitoring of trichomonad numbers, along with the implementation of treatments for affected individuals, is critical to ensure the health of Milu deer. The small sample size and the limited number of sample collection sites in this study might suggest the presence of other trichomonad species in Milu deer that have not yet been recognized. Additional regions and samples are necessary to elucidate the potential pathogenicity of trichomonads in various mucosae to assess their clinical and public health impacts.

## Conclusion

The results of this study reveal a high occurrence of trichomonads in Milu deer, identifying sample collection sites, high relative humidity, and high mean annual rainfall as significant risk factors for colonization. Trichomonads might threaten the health of Milu deer and are probably transmitted from other species. To mitigate the occurrence of trichomonad colonization, it is crucial to implement effective management strategies that minimize environmental contamination and enhance the health and welfare of Milu deer.

## Supplementary material

The supplementary material for this article can be found at https://www.parasite-journal.org/10.1051/parasite/2025015/olm. *Supplementary File 1:* Three sequences of *Hexamastix* sp. in Milu deer feces failed to upload to NCBI. *Supplementary File 2*: Phylogenetic analysis of trichomonads based on the ITS1-5.8S rRNA-ITS2 gene. Sequences were retrieved from GenBank, aligned using ClustalW, and analyzed using the MEGA 11 software. The neighbor-joining method was used to construct the trees from the Kimura-2-parameter model. Branch numbers represent percent bootstrapping values from 1,000 replicates, with values of more than 60% shown in the tree. The species identified in this study are indicated by ▲. In order to rationalize Figure 3, we present a more general phylogenetic analysis tree covering a wider range of trichomonad.
