# Supplementary material for: Genetic diversity of Trichomonads from Milu deer (Elaphurus davidianus) in China
Source: Parasite. 2025 Apr 9;32:23. doi: 10.1051/parasite/2025015 (PMC11987501; doi:10.1051/parasite/2025015)
Supplement: Supplementary file 1 — Three sequences of Hexamastix sp. in Milu deer feces failed to upload to NCBI. [file parasite-32-23-s1.pdf]

>Seq1 *Hexamastix* sp. Chinese Milu deer (*Elaphurus davidianus*) China
ATCAGTTTCGTAAAACTAGAACGAGTGATTTCACTTAAACTTATACAAACAACAACGAGCCAAAAAAT
AGACCTCATACAATGGATGTCTTGGCTCCCCCTACGATGAAGAACATGGCATAATGTGAAAAGTGACG
GGAGTTGCATACATCGTGACCGGTTCAATCTTTGAATGCATATTGCCCTCTTTGGAGGATATTATTTTGT
GTGTCCTTTCATATCAAAAATGGCTGCAAAGCTTTAAACTAATTAATTGGTTGAGGGACCACAGGGTAA
TA

>Seq2 *Hexamastix* sp. Chinese Milu deer (*Elaphurus davidianus*) China
TTCAGTTTCGTAAAACTAGAACGAGTGATTTCACTTAAACTTATACAAACAACAACGAGCCAAAAATA
GACCTCATACAATGGATGTCTTGGCTCCCCCTACGATGAAGAACATGGCATAATGTGAAAAGTGACGG
GAGTTGCATACATCGTGACCGGTTCAATCTTTGAATGCATATTGCCCTCTTTGGAGGATATTATTTTGTG
TGTCCTTTCATATCAAAAATGGCTGCAAAGCTTTAGACTAATTAATTGGTTGAGGGACCACAGGGTAAT
A

>Seq3 *Hexamastix* sp. Chinese Milu deer (*Elaphurus davidianus*) China
ATCAGTTTCGTAAAACTAGAACGAGTGATTTCACTTAAACTTATACAAACAACAACGAGCCAAAAAAT
AGACCTCATACAATGGATGTCTTGGCTCCCCCTACGATGAAGAACATGGCATAATGTGAAAAGTGACG
GGAGTTGCATACATCGTGACCGGTTCAATCTTTGAATGCATATTGCCCTCTTTGGAGGATATTATTTTGT
GTGTCCTTTCATATCAAAAATGGCTGCAAAGCTTTAAACTAATTAATTGGTTGAGGGACCACAGGGTAA
TA

Additional file 1: Three sequences failed to upload to NCBI.
