# Supplementary material for: Genetic diversity of Trichomonads from Milu deer (Elaphurus davidianus) in China
Source: Parasite. 2025 Apr 9;32:23. doi: 10.1051/parasite/2025015 (PMC11987501; doi:10.1051/parasite/2025015)
Supplement: Supplementary file 2 — Phylogenetic analysis of trichomonads based on the ITS1-5.8S rRNA-ITS2 gene. Sequences were retrieved from GenBank, aligned using ClustalW, and analyzed using the MEGA 11 software. The neighbor-joining method was used to construct the trees from the Kimura-2-parameter model. Branch numbers represent percent bootstrapping values from 1,000 replicates, with values of more than 60% shown in the tree. The species identified in this study are indicated by ▲. In order to rationalize Figure 3, we present a more general phylogenetic analysis tree covering a wider range of trichomonad. [file parasite-32-23-s2.pdf]

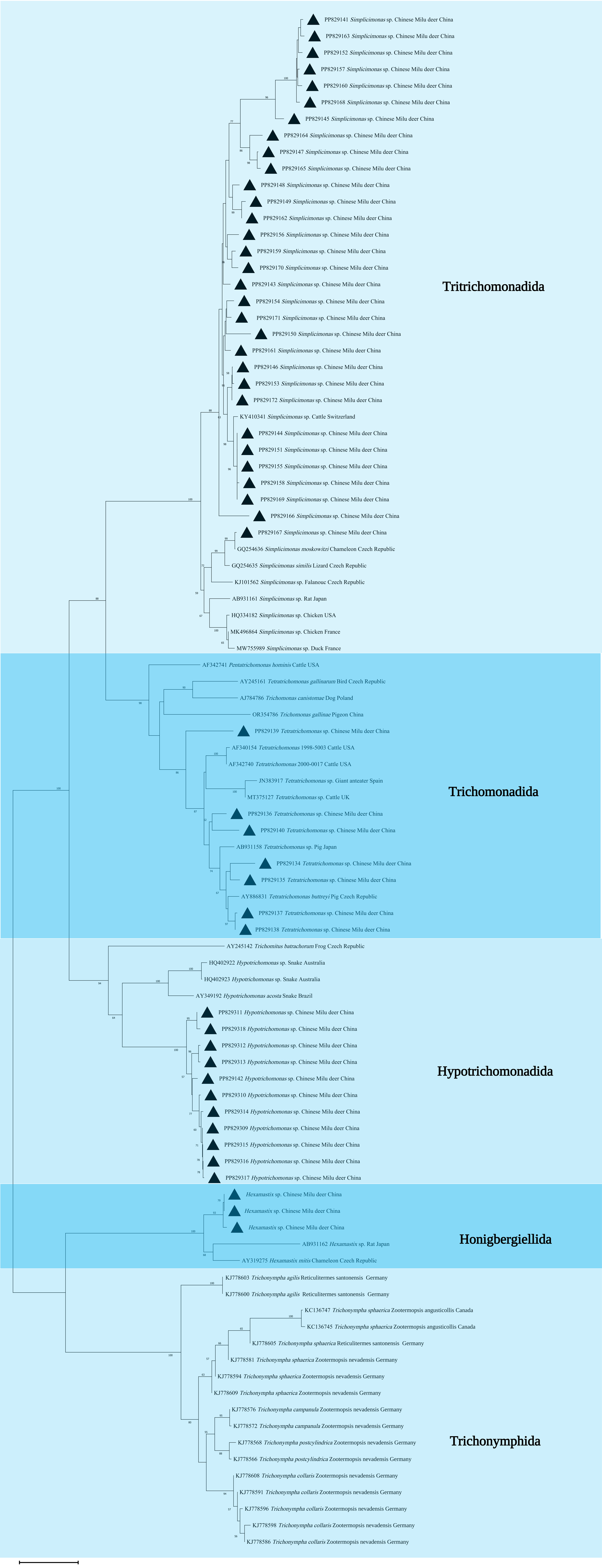

Phylogenetic analysis of trichomonads based on the ITS1-5.8S rRNA-ITS2 gene. Sequences were retrieved from GenBank, aligned using ClustalW, and analyzed using the MEGA 11 software. The neighbor-joining method was used to construct the trees from the Kimura-2-parameter model. Branch numbers represent percent bootstrapping values from 1,000 replicates, with values of more than 60% shown in the tree. The species identified in this study are indicated by ▲. In order to rationalize figure 3, we present a more general phylogenetic analysis tree covering a wider range of trichomonad.
